# Introduction to AIMS Special Issue “Obesity and Food Intake in Vulnerable Populations”

**DOI:** 10.3934/publichealth.2015.1.161

**Published:** 2015-04-01

**Authors:** Junfeng Jiao

**Affiliations:** Urban Information Lab, School of Architecture, The University of Texas at Austin, Austin, Texas, TX 78712, USA

With global obesity on the rise, the need for research concerning the complexity of risk factors is more important than ever. While the increase of the high-calorie, low-nutrient food supply correlates with rising weights, new studies across disciplines reveal the intricacy of the environmental and circumstantial phenomena that influence obesity rates and behaviors for at-risk individuals. AIMS Public Health is pleased to present this special issue entitled “Obesity and Food Intake in Vulnerable Populations” in support of this research. This edition contains two research reviews, a research study, and a brief report related to obesity and human behavior.

In the issue's first article, McGuigan and Wilkinson [1] assess whether obesity is a factor in avoiding healthcare through the examination of related journal articles published over a 22-year period, finding that perceived bias, along with other factors, contributes to obese patients evading care. Meanwhile, Vilas, Rubalcava, Becerra, and Para [2] investigate how gender dysphoria and its associated treatment contribute to obesity through hormonal therapies and lifestyle changes, advocating for the inclusion of dietary education in treatments. On the other end of the spectrum, Kjøllesdal and Holmboe-Ottesen [3] evaluate the affect of a mother's dietary choices and food intake on the prevalence of low birth weight babies. Through the analysis of studies on diet and birth weight in high-income countries, the authors found that processed food and sugar intake associate with low birth weights. Finally, Divin and Zullig [4] explore a different aspect of vulnerable populations and public health in studying the relationship between adolescent non-medical prescription drug use and suicide through responses in the National Youth Risk Behavior Survey.

The topics investigated in these articles illustrate the highly complicated and multifaceted relationships between vulnerable populations and public health outcomes, with a focus on healthcare and food intake. The theories explored provide a model for researchers seeking to demystify risk factors for and causes of obesity beyond the simple calorie-exercise balance explanation. The authors in this issue acknowledge the role that outside influences can play in healthy behaviors, and advocate for the consideration of these environmental and interactive factors by professionals in the medical establishment.
